# Improved detection of DNA-binding proteins via compression technology on PSSM information

**DOI:** 10.1371/journal.pone.0185587

**Published:** 2017-09-29

**Authors:** Yubo Wang, Yijie Ding, Fei Guo, Leyi Wei, Jijun Tang

**Affiliations:** 1 School of Computer Science and Technology, Tianjin University, Tianjin 300350, China; 2 Department of Computer Science and Engineering, University of South Carolina, Columbia, SC 29208, United States of America; 3 Tianjin University Institute of Computational Biology, Tianjin 300350, China; Huazhong University of Science and Technology, CHINA

## Abstract

Since the importance of DNA-binding proteins in multiple biomolecular functions has been recognized, an increasing number of researchers are attempting to identify DNA-binding proteins. In recent years, the machine learning methods have become more and more compelling in the case of protein sequence data soaring, because of their favorable speed and accuracy. In this paper, we extract three features from the protein sequence, namely NMBAC (Normalized Moreau-Broto Autocorrelation), PSSM-DWT (Position-specific scoring matrix—Discrete Wavelet Transform), and PSSM-DCT (Position-specific scoring matrix—Discrete Cosine Transform). We also employ feature selection algorithm on these feature vectors. Then, these features are fed into the training SVM (support vector machine) model as classifier to predict DNA-binding proteins. Our method applys three datasets, namely PDB1075, PDB594 and PDB186, to evaluate the performance of our approach. The PDB1075 and PDB594 datasets are employed for Jackknife test and the PDB186 dataset is used for the independent test. Our method achieves the best accuracy in the Jacknife test, from 79.20% to 86.23% and 80.5% to 86.20% on PDB1075 and PDB594 datasets, respectively. In the independent test, the accuracy of our method comes to 76.3%. The performance of independent test also shows that our method has a certain ability to be effectively used for DNA-binding protein prediction. The data and source code are at https://doi.org/10.6084/m9.figshare.5104084.

## Introduction

DNA-binding proteins play an important role in a variety of biomolecule functions, such as transcription, the detection of DNA damage and replication. The importance of DNA-binding proteins is facilitating the development of various methods for identifying them. Experimental methods that have been applied to identify DNA-binding proteins include filter binding assays, genetic analysis, chromatin immune precipitation on microarrays and X-ray crystallography [1, 2]. Nevertheless, these experimental methods have some disadvantages, such as expensive and time-consuming. Especially with the development of next-generation high-throughput DNA sequencing techniques [3], protein sequence data are growing rapidly. At present, it is unrealistic to use experimental methods to identify all DNA-binding proteins. Therefore, a lot of computational methods based on machine learning (ML) algorithm or statistical model [4–6] are used to reduce the cost of resources. In order to further facilitate the calculation process, there are some web servers have been developed to generate feature vectors of DNA, RNA or protein sequences, such as a web-server called Pse-in-One [7]. In recent years, computational methods based on machine learning (ML) algorithms have become more and more popular because of their promising performance. According to various feature information, the ML-based approachs are mainly composed of structure information-based [8–18] and sequence information-based method [1, 2, 19–35].

The structural features of proteins are closely related to the functions, and therefore predictors based on the structural information can achieve better performance of DNA-binding protein identification. Nimrod et al. [18] trained a random forest classifier using the average surface electrostatic potentials, dipole moments and cluster-based amino acid conservation patterns of the protein. Ahmad et al. [10] developed a neural network classifier based on the net charge, electric dipole moment and quadrupole moment tensors of the protein. Bhardwaj et al. [13] made use of SVM classifier and three features, including surface and overall composition, overall charge and positive potential surface patches. Some structure-based methods also have the participation of sequence information. For example, Szilágyi and Skolnick [17] extracted feature vectors from the following three perspectives: the relative proportions of certain amino acids, the asymmetry of the spatial distribution of certain other amino acids and the dipole moment of the molecule. However, a large number of proteins can’t be known with the structural information, so structure-based predictors can only be applied to a small portion of the whole protein database.

In contrast, sequence information is easier to extract and more convenient to use. We can extract multiple sequence-based features, such as physicochemical properties [20, 36], dipeptide composition [24, 30] and the amino acid composition [21]. Cai and Lin [20] trained SVM classifiers using protein’s amino acid composition, limited range correlation of hydrophobicity and solvent accessible surface area of the protein. Yu et al. [21] developed the binary classifications for rRNA-, RNA-, DNA-binding proteins by feeding these features (being extracted from protein sequence amino acid compositions and physicochemical properties) into the SVM classifier. Liu et al. [36] extracted feature vectors from three sequence features, including overall amino acid composition, pseudo amino acid composition and physicochemical distance transformation. Some researchers also incorporated evolutionary information generated by PSI-BLAST [37] into sequence-based methods to improve prediction performance. For instance, Kumar et al. [38] were the first to use evolutionary information to identify DNA-binding proteins and developed a SVM classifier called DNAbinder. Some similar methods, for example the method of Ho et al. [39], were also proposed to identify DNA-binding proteins. Their results showed that evolutionary information can significantly improve the performance, so evolutionary information is useful in the identification of DNA binding proteins. Liu et al. [25] proposed a predictor called iDNAPro-PseAAC, which incorporates evolutionary information and the pseudo amino acid composition (PseAAC). The method of Waris et al. [28] used features extracted from dipeptide composition, split amino acid composition and position specific scoring matrix (PSSM) to train multiple classifiers and found the classifier that achieved the best predicte performance.

As described above, the feature extraction algorithms determine whether protein sequences can be expressed completely by feature vectors. In order to obtain a satisfactory performance, we should select feature extraction algorithms carefully. In this paper, we innovatively combine the 1040-dimension feature vector named PSSM-DWT, the 100-dimension feature vector named PSSM-DCT and the 200-dimension feature vector named NMBAC to predict DNA-binding proteins. Discrete Wavelet Transform (DWT) and Discrete Cosine Transform (DCT) can be used to obtain the effective information by compressing the PSSM matrix. Also, we extract the 200-dimension feature vector according to six physicochemical properties. Then, these features are fed into the training SVM model for predicting DNA-binding proteins. We evaluate our method by three datasets, namely PDB1075, PDB594 and PDB186. The first two datasets are used in Jackknife test and the last dataset, PDB186, is used for independent testing. The results demonstrate the effectiveness of our method in identifying DNA-binding proteins.

## Materials and methods

**Fig 1:**
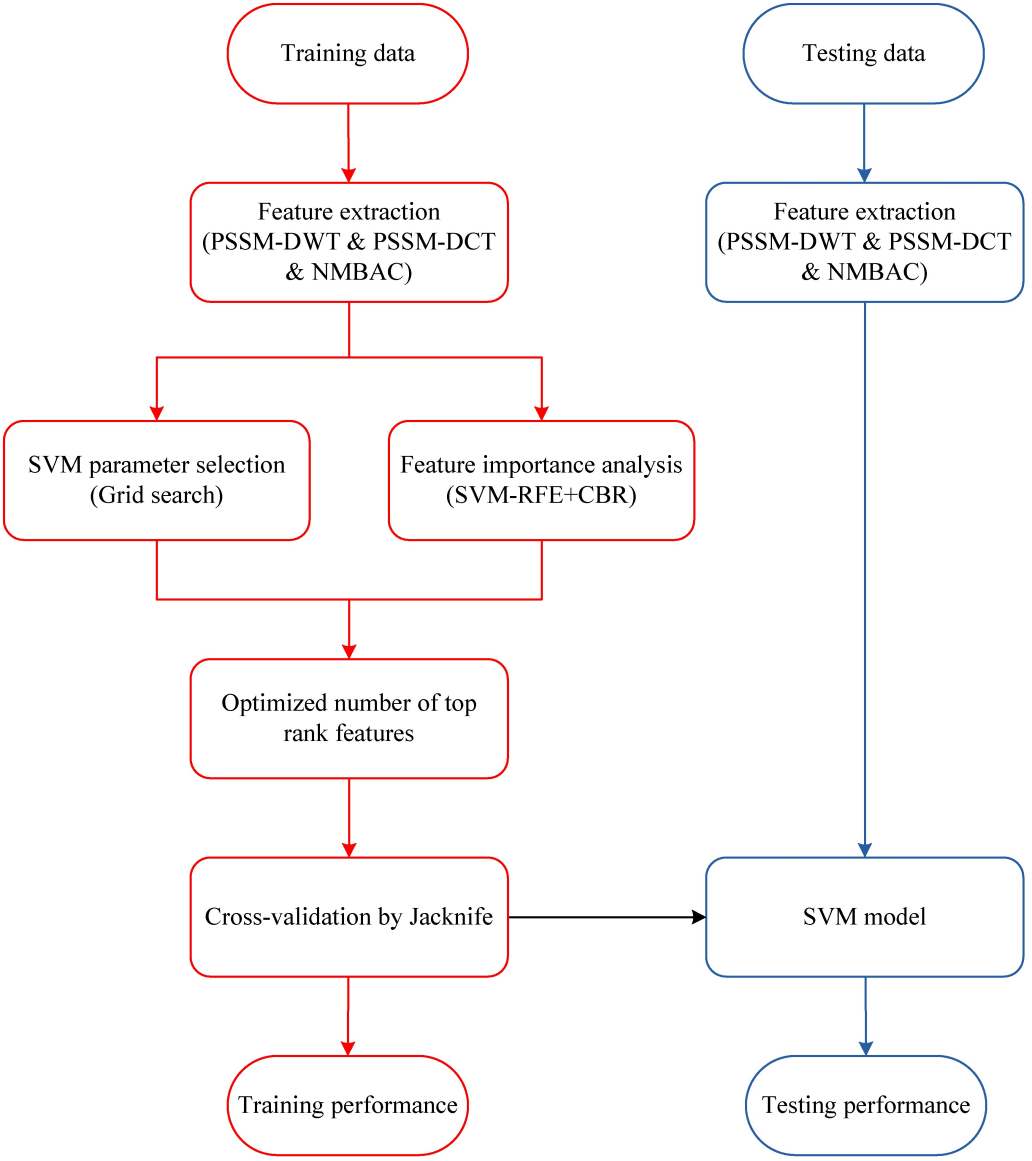
The framework of our method.

In order to illustrate the overall process, the framework of our method is presented in Fig 1. In the training phase, we extract two features (PSSM-DWT and PSSM-DCT) from the PSSM matrix, and extract NMBAC feature from six physicochemical properties. The prediction model is obtained by feeding these features into the SVM classifier. In the prediction phase, we use the same feature representation algorithm to describe the predictive protein sequence, then use the training SVM model for DNA-binding protein prediction.

### Datasets

In the present study, we apply three benchmark datasets to evaluate our approach, namely PDB1075, PDB594 and PDB186. These DNA-binding proteins are selected from Protein Data Bank (http://www.rcsb.org/pdb/home/home.do). The protein sequences, which are less than 50 amino acids or contain character “X”, must be removed. We should ensure that no sequence has more than 25% similarity with any other sequences. Concretely, the PDB1075 dataset, constructed by Liu et al. [40], has 525 DNA-binding proteins and 550 DNA-non-binding proteins. The PDB594 dataset, edited by Lou et al. [2], is made up of 297 DNA-binding proteins and 297 DNA-non-binding proteins. These two datasets are applied for Jackknife test. The PDB186 dataset for independent test is also derived from the paper of Lou et al. [2], and contains 93 DNA-binding proteins and 93 DNA-non-binding proteins.

### Evolutionary features

#### Position specific scoring matrix

Position Specific Scoring Matrix (PSSM) generated by PSI-BLAST [37] (BLAST+ [41] options: -num_iterations 3 -db nr -inclusion_ethresh 0.001) stores the evolutionary information of a protein sequence. Suppose the length of a protein sequence is *L* (*L* amino acids), the size of the PSSM for this protein is *L* × 20 (*L* rows and 20 columns). The form of this matrix is as follows:

$$\begin{array}{c} PSSM_{original}={\left[\begin{array}{cccc} P_{1,1} & P_{1,2} & \cdots & P_{1,20}\\ P_{2,1} & P_{2,2} & \cdots & P_{2,20}\\ \vdots & \ddots & \vdots & \vdots \\ P_{L,1} & P_{L,2} & \cdots & P_{L,20} \end{array}\right]}_{L\times 20} \end{array}$$ (1)

The formula for each element *PSSM*_*original*_(*i*, *j*) is as follows:

$$\begin{array}{c} PSSM_{original}\left(i,j\right)=\sum_{k=1}^{20}\;\omega \left(i,k\right)\times D\left(k,j\right),i=1,\ldots ,L,j=1,\ldots ,20 \end{array}$$ (2)

where *ω*(*i*, *k*) is the frequency of *k*-th amino acid type at the position *i*, *D*(*k*, *j*) is the rate of mutation from the *k*-th amino acid to the *j*-th amino acid in a protein sequence from Dayhoff’s mutation matrix (substitution matrix). The larger values of substitution matrix indicate more strongly conserved positions; otherwise, the reverse.

#### Discrete Cosine Transform

We use the Discrete Cosine Transform (DCT) [42], which is widely used in data compression to compress PSSM and retain a portion of the compressed PSSM as feature vectors. The DCT is a linear separable transformation and can change the distribution of information density from evenly to unevenly. After compression, we should retain the low frequency part of PSSM, because the low frequency section contains more information than the high frequency section. In this work, 2 dimensions DCT (2D-DCT) is used to compress PSSM. Given an input matrix *Mat* = *PSSM*_*original*_ ∈ ℜ^*L*×20^, the corresponding conversion formula is as follows:

$$\begin{array}{c} DCT\left(i,j\right)=\alpha_{i}\alpha_{j}\;\sum_{m=0}^{M-1}\;\sum_{n=0}^{N-1}\;Mat\left(m,n\right)cos\frac{\pi (2m+1)i}{2M}\times cos\frac{\pi (2n+1)j}{2n} \end{array}$$ (3a)

$$\begin{array}{c} \alpha_{i}=\{\begin{array}{c} \sqrt{1/M},\;i=0\\ \sqrt{2/M},\;1\leq i\leq M-1 \end{array} \end{array}$$ (3b)

$$\begin{array}{c} \alpha_{j}=\{\begin{array}{c} \sqrt{1/N},\;j=0\\ \sqrt{2/N},\;1\leq j\leq N-1 \end{array} \end{array}$$ (3c)

where 0 ≤ *i* < *M*, 0 ≤ *j* < *N*.

According to the above formula for compression, the part that contains most of the information (low frequency section) is distributed in the upper left corner of the compressed PSSM. In the end, we retain the first 100 coefficients as PSSM-DCT feature.

#### Discrete Wavelet Transform

**Fig 2:**
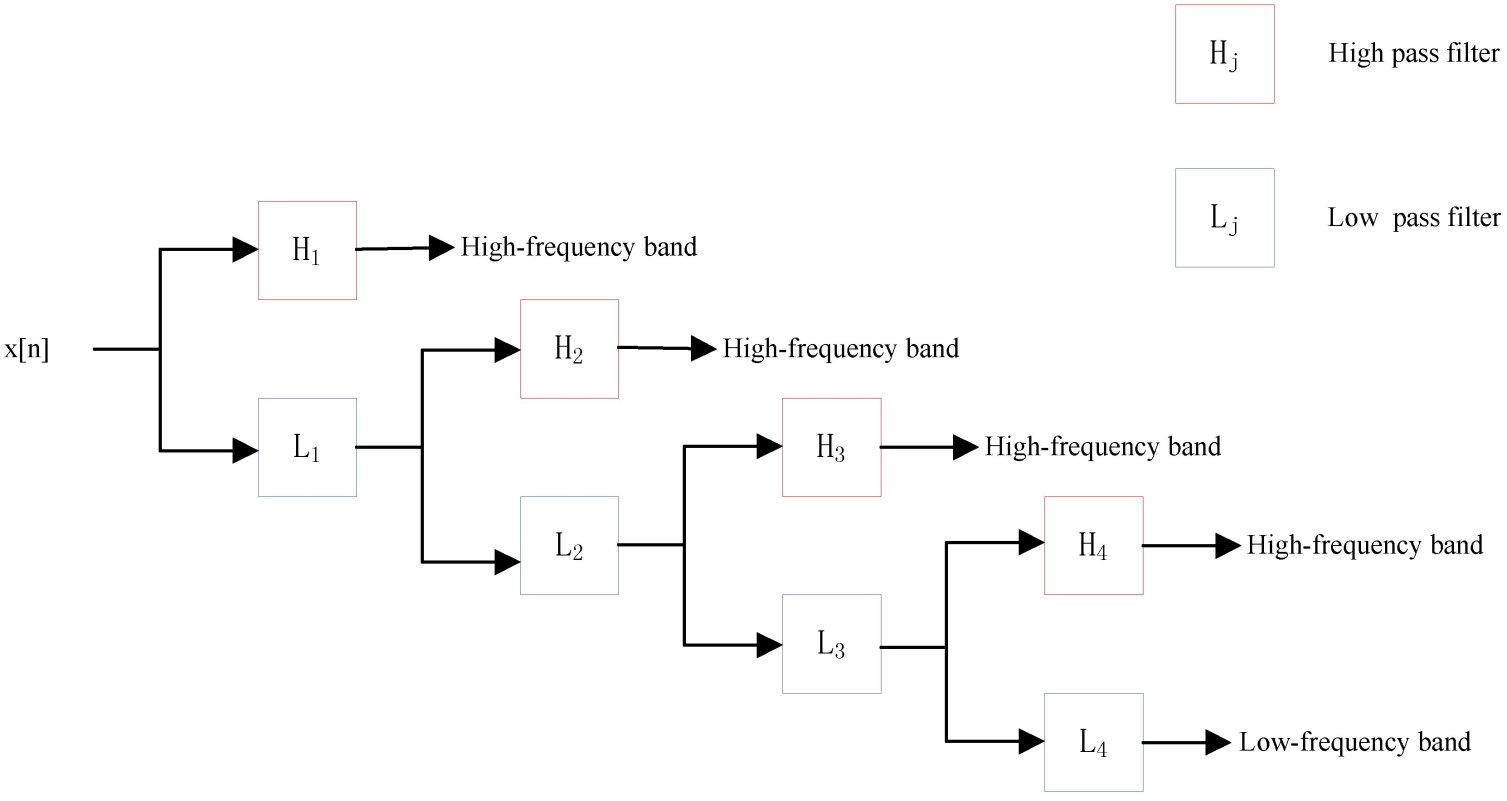
Schematic diagram of a 4-level DWT.

The Wavelet Transform (WT) is defined as the projection of a signal *f*(*t*) onto the wavelet function:

$$\begin{array}{c} T\left(a,b\right)=\sqrt{1/a}\;\int_{o}^{t}\;f\left(t\right)\psi \;(\frac{t-b}{a})\;d_{t} \end{array}$$ (4)

where *a* is a scale variable and *b* is a translation variable. $\psi \;(\frac{t-b}{a})$ is the analyzing wavelet function. *T*(*a*, *b*) is the transform coefficients which are found for both specific locations on the signal and specific wavelet periods. Discrete Wavelet Transform (DWT) can decompose the amino acid sequences into coefficients at different dilations and then remove the noise component from the profiles. Nanni et al. [43, 44] proposed an efficient algorithm to perform DWT by assuming that the discrete signal *f*(*t*) is *x*[*n*], where *N* is the length of discrete signal.

$$\begin{array}{c} y_{j,low}\left[n\right]=\sum_{k=1}^{N}\;x\left[k\right]g\left[2n-k\right] \end{array}$$ (5a)

$$\begin{array}{c} y_{j,high}\left[n\right]=\sum_{k=1}^{N}\;x\left[k\right]h\left[2n-k\right] \end{array}$$ (5b)

where *g* is low pass filter and *h* is high pass filter. *y*_*low*_[*n*] is the approximate coefficient (low-frequency components) of the signal. *y*_*high*_[*n*] is the detailed coefficient (high-frequency components). This decomposition is repeated to further increase the frequency resolution and the approximation coefficients decomposed with high and low pass filters and then down-sampled. With the increase of decomposition level *j*, more detailed characteristics of the signal can be observed. Inspired by Nanni’s work [43, 44], we use 4-level DWT and calculate the maximum, minimum, mean and standard deviation values of different scales (4 levels of both low and high-frequency coefficients). Because of the high-frequency components are more noisy and hence only the low-frequency components are more important, we also extract the first five discrete cosine coefficients from the approximation coefficients. The schematic diagram of a 4-level DWT is shown in Fig 2.

The *Mat* = *PSSM*_*original*_ ∈ ℜ^*L*×20^ has 20 columns. So, the PSSM consists of 20 types of discrete signals (*L* lengths). At last, we use above 4 levels DWT to analysis these discrete signals of PSSM (each column) and extract the PSSM-DWT feature from PSSM of protein.

### Sequence features

#### Normalized Moreau-Broto Autocorrelation

We use the Normalized Moreau-Broto Autocorrelation(NMBAC) to extract sequence features from six physicochemical properties for improving the predicte performance. The NMBAC is proposed by Feng at al. [45] for the prediction of membrane protein types. Each physicochemical propertie of 20 amino acid have corresponding values and a protein sequence can be replaced by a vector of physicochemical property values. In our work, the six physicochemical properties are hydrophobicity (H), volumes of side chains of amino acids (VSC), polarity (P1), polarizability (P2), solvent-accessible surface area (SASA) and net charge index of side chains (NCISC) of amino acid, respectively. The physicochemical propertie values of 20 amino acids are shown in Table 1. Before we use these values to represent amino acids, they must be normalized to zero mean and unit standard deviation (SD) as follows:

$$\begin{array}{c} P_{i,j}^{\prime }=\frac{P_{i,j}-P_{j}}{S_{j}}\;\left(i=1,2,\ldots ,20;\;j=1,2,\ldots ,6.\right) \end{array}$$ (6)

where *P*_*i*,*j*_ is the value of descriptor *j* for amino acid type *i*, *P*_*j*_ is the mean over 20 amino acids of descriptor value *j*, and *S*_*j*_ is the corresponding SD.

**Table 1:** Original values of six physicochemical properties of 20 amino acid types.

Amino acid	H	VSC	P1	P2	SASA	NCISC
A	0.62	27.5	8.1	0.046	1.181	0.007187
C	0.29	44.6	5.5	0.128	1.461	-0.03661
D	-0.9	40	13	0.105	1.587	-0.02382
E	-0.74	62	12.3	0.151	1.862	0.006802
F	1.19	115.5	5.2	0.29	2.228	0.037552
G	0.48	0	9	0	0.881	0.179052
H	-0.4	79	10.4	0.23	2.025	-0.01069
I	1.38	93.5	5.2	0.186	1.81	0.021631
K	-1.5	100	11.3	0.219	2.258	0.017708
L	1.06	93.5	4.9	0.186	1.931	0.051672
M	0.64	94.1	5.7	0.221	2.034	0.002683
N	-0.78	58.7	11.6	0.134	1.655	0.005392
P	0.12	41.9	8	0.131	1.468	0.239531
Q	-0.85	80.7	10.5	0.18	1.932	0.049211
R	-2.53	105	10.5	0.291	2.56	0.043587
S	-0.18	29.3	9.2	0.062	1.298	0.004627
T	-0.05	51.3	8.6	0.108	1.525	0.003352
V	1.08	71.5	5.9	0.14	1.645	0.057004
W	0.81	145.5	5.4	0.409	2.663	0.037977
Y	0.26	117.3	6.2	0.298	2.368	0.023599

For each physicochemical property, a protein can be represented by a vector composed of normalized physicochemical property values. NMBAC [45] is obtained by inputting these vectors into the following formula:

$$\begin{array}{c} NMBAC_{lag,j}=\frac{1}{\left(n-lag\right)}\;\sum_{i=1}^{n-lag}\;\left(X_{i,j}\times X_{i+lag,j}\right) \end{array}$$ (7a)

$$\begin{array}{c} \left(i=1,2,\ldots ,n-lag;\;j=1,2,\ldots ,6.\right) \end{array}$$ (7b)

where *j* represents one descriptor of six descriptor, *i* is the position in protein sequence *X*, *n* is the length of the protein sequence and *lag* is the sequential distance between one residue and another, a certain number of residues away (*lag* = 1, 2, …, *lg*, *lg* is a parameter determined by an optimization procedure to be described).

According to Guo’s work [46], we define the optimal value of *lag* from 1 to 30. For each protein sequence, we can obtain 30 × 6 = 180 dimensional feature vector. We also add the frequency of 20 amino acids appearing on this sequence to the feature vector. Finally, we can get the 30 × 6 + 20 = 200 dimensional feature vector for a protein sequence.

### Classification and feature selection

After feature extraction procedure, all samples in benchmark datasets are converted into numerical feature vectors with the same dimension. The feature space of each protein sequence is composed of PSSM-DWT, PSSM-DCT and NMBAC features. By removing noisy and redundant features from the original feature space (PSSM-DWT + PSSM-DCT + NMBAC), feature selection alleviates the overfitting and improves the performance. In order to reduce feature abundance and computation complexity, we use the Support Vector Machine Recursive Feature Elimination and Correlation Bias Reduction (SVM-RFE+CBR) [47] to select an optimal feature subset. SVM-RFE+CBR is proposed by incorporating the CBR strategy into the feature elimination procedure: (1) less prone to overfitting; (2) able to make full use of the training data; (3) much faster, especially on a lot of candidate features. As a result, it has been successfully applied in many problems, especially in gene selection [48–50]. We can obtain the output of SVM-RFE+CBR with a ranked feature list. Feature selection is achieved by choosing a group of top-ranked features. The ranking criterion of SVM-RFE+CBR is closely related to the SVM model.

#### Support Vector Machine

Support Vector Machine (SVM) developed by Vapnik [51] is a classification and regression paradigm. In the process of using SVM, samples labeled positive or negative are projected into a high dimensional feature space using a kernel, and the hyper plane in the feature space is optimized to maximize the margin of positive and negative samples. There are some biological problems for example prediction of protein-protein interactions [46, 52–56], homology detection [57], and analysis of gene expression data [58] that can used SVM to solve. Given a training dataset of instance-label pairs {*x*_*i*_, *γ*_*i*_}, *i* = 1, 2, …, *N* with input data *x*_*i*_ ∈ *R*^*n*^ and output labels *γ*_*i*_ ∈ {+1, −1}, the classification decision function implemented by SVM is represented in the following equation:

$$\begin{array}{c} \gamma \left(x\right)=sign[\sum_{i=1}^{N}\;\gamma_{i}\alpha_{i}\cdot K\left(x,x_{i}\right)+b] \end{array}$$ (8)

where the coefficient *α*_*i*_ is obtained by solving the following convex Quadratic Programming (QP) problem:

$$\begin{array}{c} \text{Maximize}\;\sum_{i=1}^{N}\;\alpha_{i}-\frac{1}{2}\;\sum_{i=1}^{N}\;\sum_{j=1}^{N}\;\alpha_{i}\alpha_{j}\cdot \gamma_{i}\gamma_{j}\cdot K\left(x_{i},x_{j}\right) \end{array}$$ (9a)

$$\begin{array}{c} \text{s.t.}\;0\leq \alpha_{i}\leq C \end{array}$$ (9b)

$$\begin{array}{c} \sum_{i=1}^{N}\;\alpha_{i}\gamma_{i}=0,i=1,2,\ldots ,N \end{array}$$ (9c)

where *x*_*j*_ is called pupport vector only if the corresponding *α*_*j*_ > 0, *C* is a regularization parameter that controls the tradeoff between margin and misclassification error.

Under most circumstances, *K*(*x*_*i*_, *x*_*j*_) = *exp*(−*γ*‖*x*_*i*_ − *x*_*j*_‖^2^), called the Radial Basis Functions (RBF) kernel, has better boundary response, and most high-dimensional data are approximated by Gaussian-like distributions. We implemented a SVM model using LIBSVM [59] with the radial basis functiona (http://www.csie.ntu.edu.tw/∼cjlin/libsvm/).

## Results and discussion

We preform our method on three datasets for predicting DNA-binding protein. In the Jackknife test, we apply our method on the PDB1075 and PDB594 datasets to analyze the effectiveness of feature extraction and feature selection, the performance of our method is also compared with other methods. In the independent test, our prediction model is tested on the independent dataset PDB186 and compared with the results of other methods.

### Measurements

We use the Jackknife test to analyze the quality of predictor constructed by our method. Because of the effectiveness of Jackknife test, it is widely used to test the function of predictor(eg., [26, 60]). In the Jackknife test, we use every sample of the benchmark dataset as test dataset one by one, and the rest of the samples are used to train predictor.

In addition, We employ four mearsures which are also used in other methods to evaluate the performance of our method, including Accuracy (ACC), Sensitivity (SN), Specificity (SP), and Mathew’s correlation coefficient (MCC). Their formulas are listed blelow:

$$\begin{array}{c} ACC=\frac{TP+TN}{TP+FP+TN+FN} \end{array}$$ (10a)

$$\begin{array}{c} SN=\frac{TP}{TP+FN} \end{array}$$ (10b)

$$\begin{array}{c} SP=\frac{TN}{TN+FP} \end{array}$$ (10c)

$$\begin{array}{c} MCC=\frac{TP\times TN-FP\times FN}{\sqrt{\left(TP+FN)\times (TN+FP)\times (TP+FP)\times (TN+FN\right)}} \end{array}$$ (10d)

where TP is the number of true positive, TN is the number of true negative, FP is the number of false positive, and FN is the number of false negative.

### Parameter optimization

**Fig 3:**
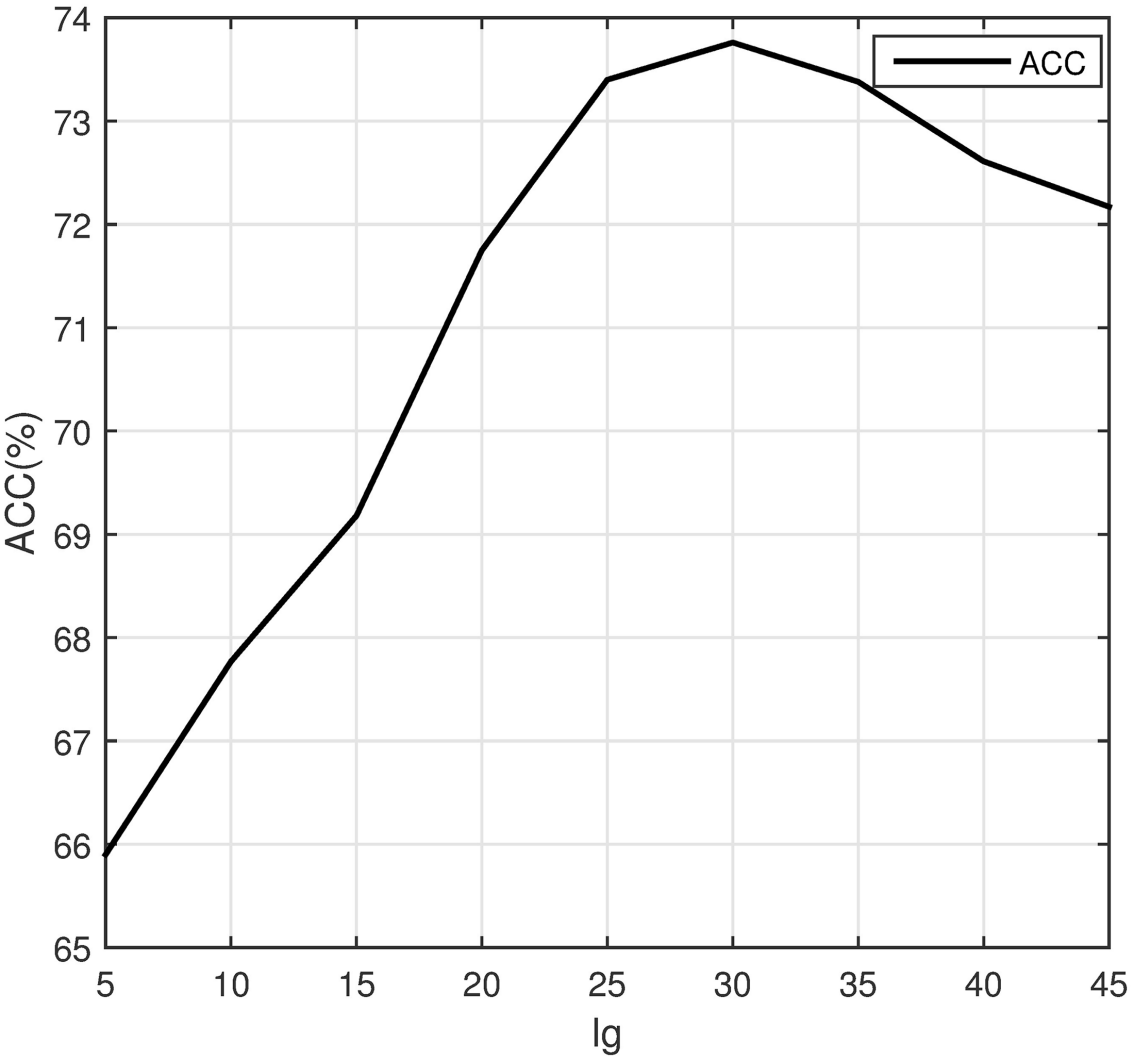
The accuracy of different lg values on PDB1075 (Five-fold cross validation).

**Fig 4:**
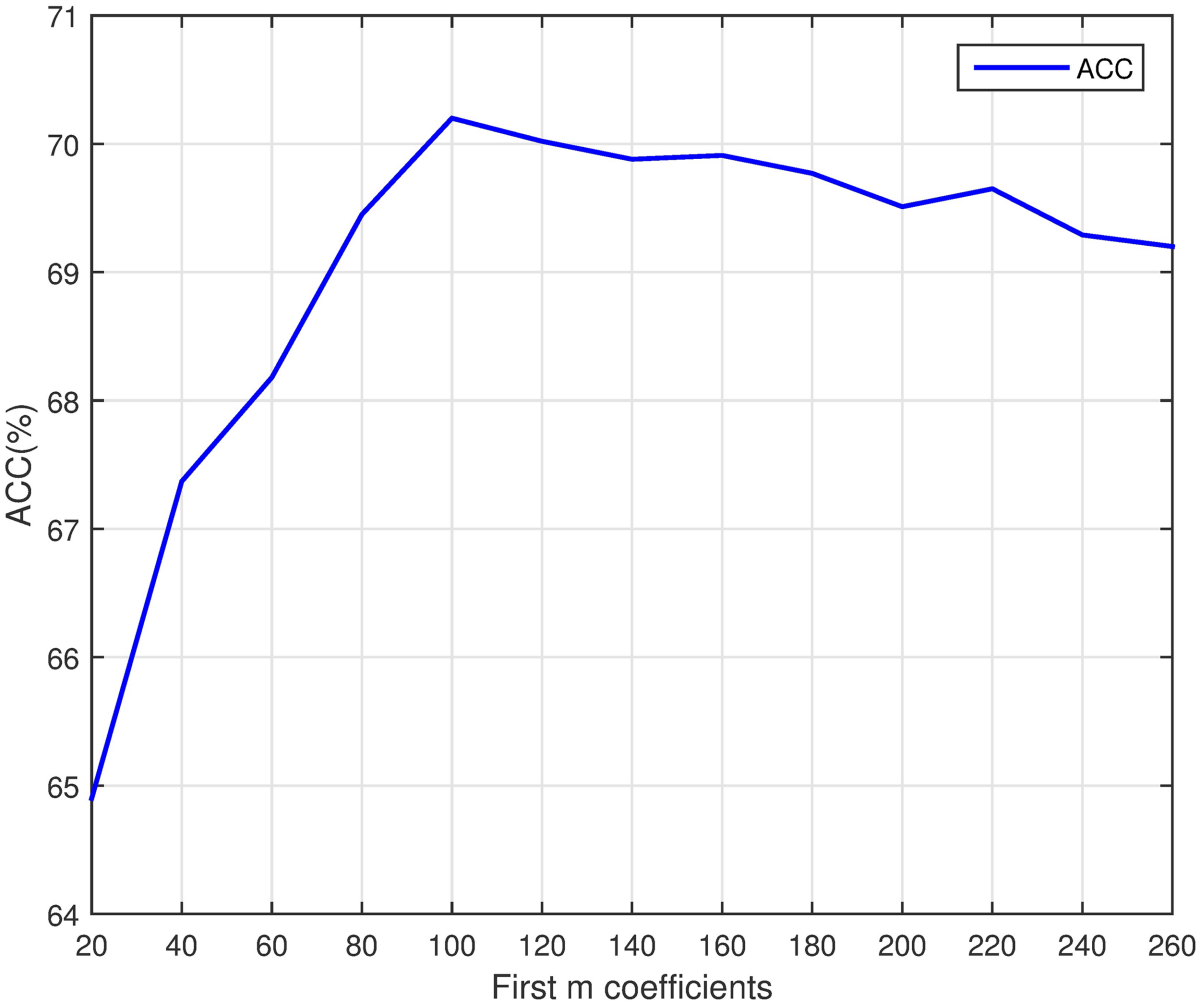
The accuracy of different m values on PDB1075 (Five-fold cross validation).

To select the optimal parameters of feature NMBAC and PSSM-DCT, we test the predictive performance for different parameters (NMBAC with different value of lg and PSSM-DCT with different first m coefficients) via a five-fold cross validation. To get the optimal lg, we evaluate values of lg from 5 to 45 (with a step of 5). The results of the prediction on PDB1075 dataset are shown in Fig 3. The ACC of prediction is increasing, when the value of lg is between 5 and 30. After that, the value of ACC is falling. Different value of m may lead to different performance, we test different values of m from 20 to 260 (with a step of 20). The curve of ACC is shown in Fig 4. The value of ACC is rising, when m increases from 20 to 100. But it slightly declines, when m is between 100 and 260. Obviously, PSSM-DCT with m less than 100 (lg less than 30 for NMBAC) would lose some effective features and larger values may introduce noise. Thus, we select lg as 30 (NMBAC) and m as 100 (PSSM-DCT) in our experiments.

### Benchmark dataset—PDB1075

#### Performance of different feartures

**Fig 5:**
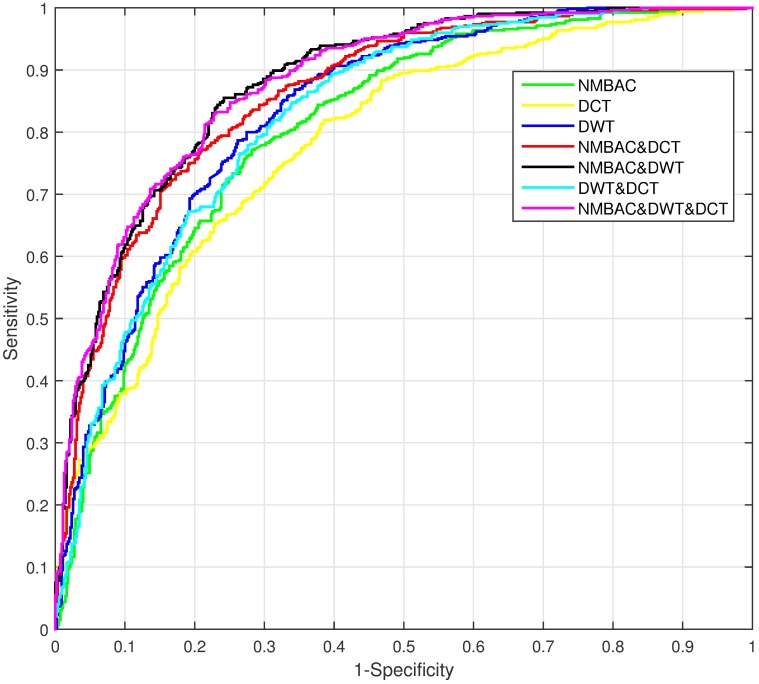
The AUROC comparison of seven feature combinations through Jackknife cross-validation on PDB1075 dataset.

We extract three features from the benchmark dataset (PDB1075), namely PSSM-DWT, PSSM-DCT and NMBAC. We need to find a combination of features to achieve the best performance, and analyze the most important feature to get the good prediction. The performance of different feartures by Jackknife test is shown in Table 2. The combination of NMBAC, PSSM-DCT and PSSM-DWT achieves the highest ACC (0.7926), MCC (0.5853), SN (0.8000) and second highest SP (0.7855). In order to obtain the importance of each feature, we compare the AUROC of seven feature combinations obtained by Jackknife cross-validation on PDB1075 dataset, shown in Fig 5. We can see that the highest contribution to the predicton performance is PSSM-DWT, followed by NMBAC, yet the PSSM-DCT is the lowest one. These information show that each feature is useful in prediction of DNA-binding proteins and the combination of three features can achieve the best performance, but the PSSM-DCT feature is not as effective as the other two features.

**Table 2:** The performance of different features on PDB1075 dataset (Jackknife test evaluation).

Feature	ACC(%)	MCC	SN(%)	SP(%)
NMBAC	74.05	0.4836	77.90	70.36
PSSM-DCT	70.60	0.4117	66.86	74.18
PSSM-DWT	75.07	0.5010	73.33	76.73
NMBAC+PSSM-DCT	78.05	0.5606	77.14	78.91
NMBAC+PSSM-DWT	78.70	0.5740	79.24	78.18
PSSM-DWT+PSSM-DCT	73.77	0.4752	73.52	74.00
NMBAC+PSSM-DWT+PSSM-DCT	79.26	0.5853	80.00	78.55

#### Performance after feature selection

**Fig 6:**
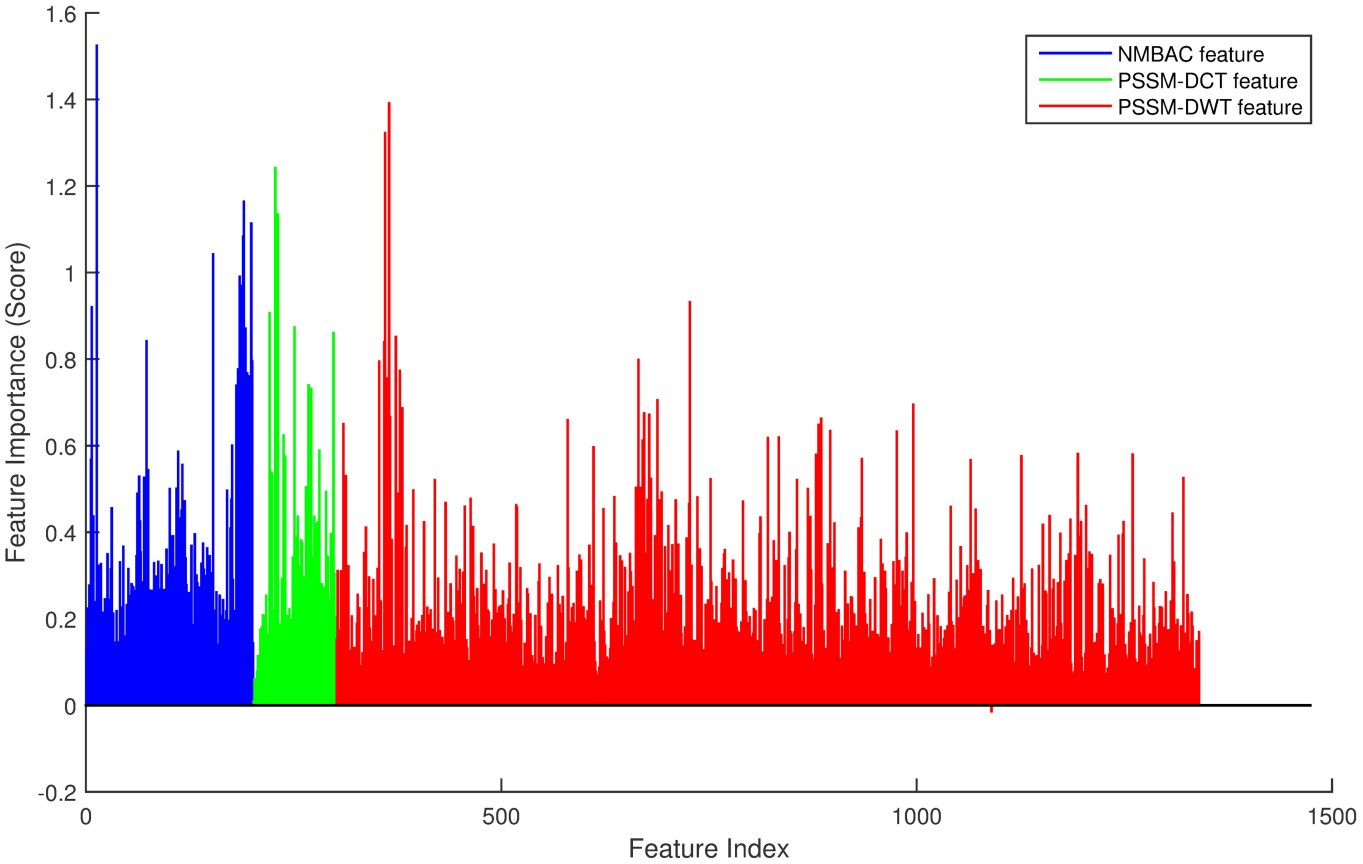
The feature score through SVM-RFE+CBR on the dataset of PDB1075. The x-axis represents the feature index.

**Fig 7:**
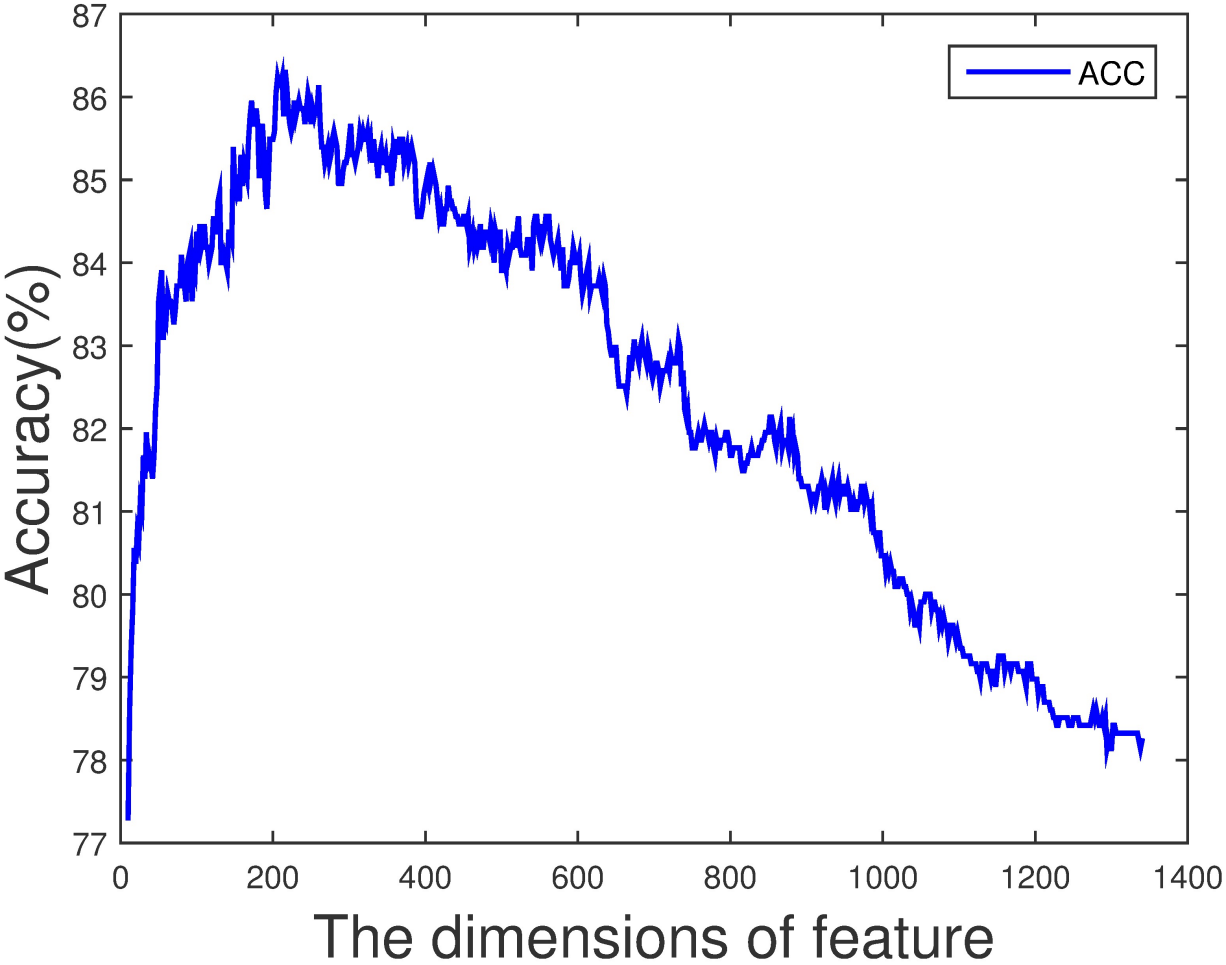
The accuracy of different dimension features on PDB1075 dataset (Jackknife test evaluation).

**Fig 8:**
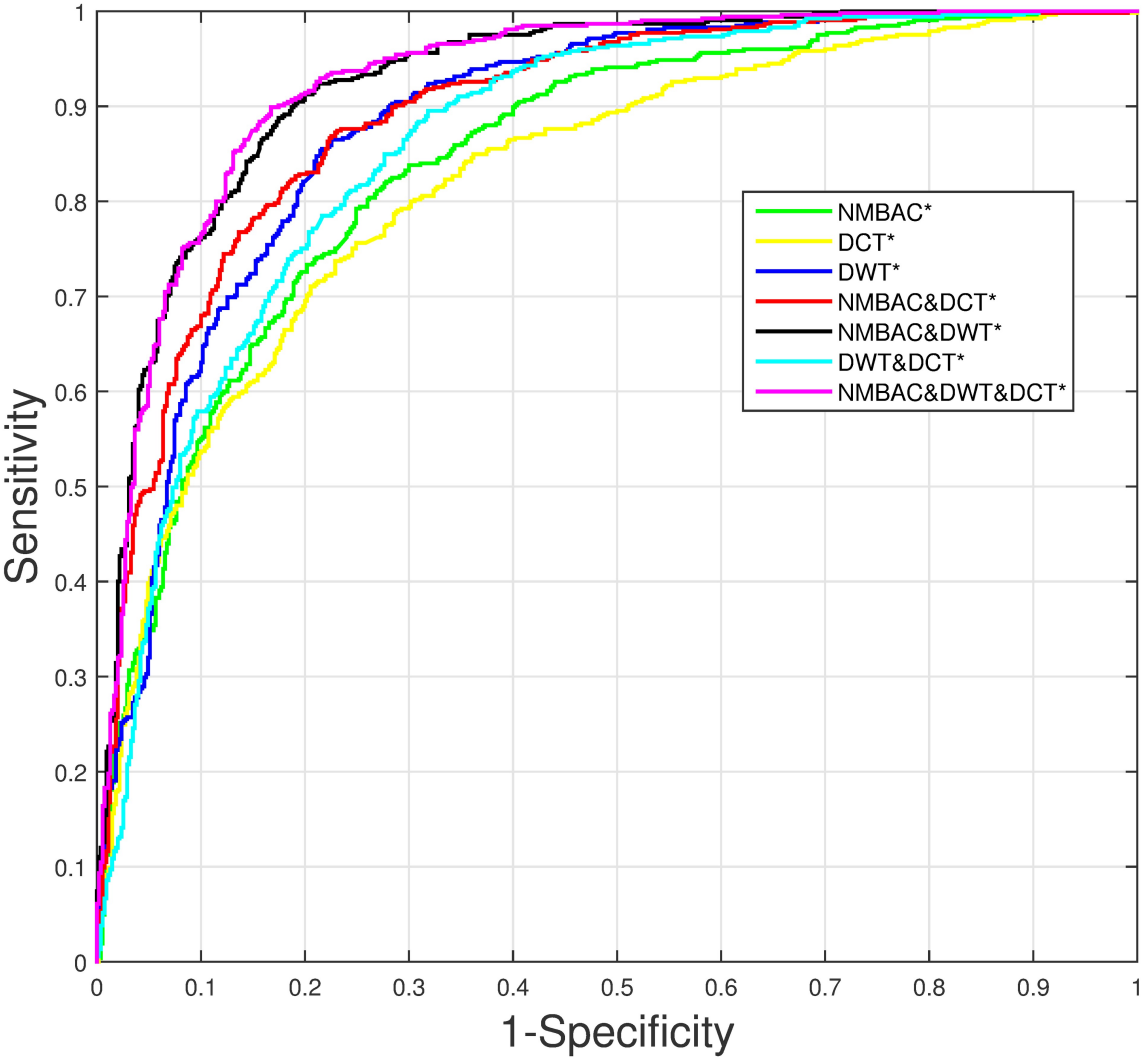
The AUROC comparison of seven feature combinations through Jackknife cross-validation on PDB1075 dataset. * means this feature combination has employed feature selection.

In order to improve the performance on PDB1075 dataset, we remove the noisy and redundant features from the original feature space by SVM-RFE+CBR [47]. Consider the combination of three features, first, we obtain a ranked feature list as shown in Fig 6. NMBAC, PSSM-DCT and PSSM-DWT are divided into three intervals: [1, 200], [201, 300] and [301, 1340]. Then the accuracy of different dimension features by Jackknife test can be seen in Fig 7 and we can find that the best accuracy can be achieved when we select the first 216-dimension features, these features can be obtained from the ranked feature list: 77 features in the interval [1, 200], 22 features in the interval [201, 300] and 117 features in the interval [301, 1340]. The feature selection is applied to each feature combination, and we can obtain the feature dimension with the best performance of each feature combination. As in the previous section, we also analyze the importance of each feature according to the AUROC comparison of seven feature combinations after feature selection, shown in Fig 8. We get the same result that NMBAC and PSSM-DWT are more effective than PSSM-DCT in the prediction of DNA-binding protein.

For the results of the previous section, We obtain new results after feature selection which be shown in Table 3. We also find that the combination of three features achieves the best performance and has obviously exceeded the performance without feature selection. It reaches the highest value on all metrics: ACC(0.8623), MCC(0.7250), SN(0.8743) and SP(0.8509). These results strongly demonstrate that feature selection can significantly improve the predict performance.

**Table 3:** The performance of different features after feature selection on PDB1075 dataset (Jackknife test evaluation).

Feature	ACC(%)	MCC	SN(%)	SP(%)
NMBAC*	76.09	0.5218	76.19	76.00
PSSM-DCT*	74.60	0.4928	76.19	73.09
PSSM-DWT*	81.02	0.6213	82.86	79.27
NMBAC+PSSM-DCT*	81.40	0.6276	80.38	82.36
NMBAC+PSSM-DWT*	84.93	0.6987	85.52	84.36
PSSM-DWT+PSSM-DCT*	78.33	0.5664	78.29	78.36
NMBAC+PSSM-DWT+PSSM-DCT*	86.23	0.7250	87.43	85.09

* means this feature combination has employed feature selection.

#### Comparision with existing methods

The performance of our method on PDB1075 dataset is compared with other existing methods, including iDNA-Prot|dis [40], iDNA-Prot [61], DNA-Prot [19], PseDNA-Pro [36], DNAbinder [38], iDNAPro-PseAAC [25], Kmer1+ACC [23] and Local-DPP [62]. The performance of different methods by Jackknife test is displayed in Table 4. We can find that four mearsures evaluated by our method are significantly higher than the evaluated mearsures of other methods. The ACC, MCC, SN and SP values of our method are improved by 7.03%, 0.13, 2.63% and 4.73%, respectively, compared with other methods.

**Table 4:** The performance of our method and other existing methods on PDB1075 dataset (Jackknife test evaluation).

Methods	ACC(%)	MCC	SN(%)	SP(%)
IDNA-Prot|dis	77.30	0.54	79.40	75.27
PseDNA-Pro	76.55	0.53	79.61	73.63
IDNA-Prot	75.40	0.50	83.81	64.73
DNA-Prot	72.55	0.44	82.67	59.76
DNAbinder(dimension = 400)	73.58	0.47	66.47	80.36
DNAbinder(dimension = 21)	73.95	0.48	68.57	79.09
iDNAPro = PseAAC	76.56	0.53	75.62	77.45
Kmer1+ACC	75.23	0.50	76.76	73.76
Local-DPP(n = 3,λ = 1)	79.10	0.59	84.80	73.60
Local-DPP(n = 2,λ = 2)	79.20	0.59	84.00	74.50
Our method	86.23	0.73	87.43	85.09

### Benchmark dataset—PDB594

We compare the performance of our method with several classifiers applied to Lou’s method [2] on the benchmark dataset (PDB594), shown in Table 5. Our method achieve the highest ACC of 86.2%, MCC of 0.724, SN of 87.2% and SP of 85.2%. The ACC, MCC, SN and SP values are improved by 5.7%, 0.114, 1.7% and 7.1%, respectively. This represents the effectiveness of our method for identifying DNA-binding proteins.

**Table 5:** The performance of our method and other existing methods on PDB594 dataset (Jackknife test evaluation).

Methods	ACC(%)	MCC	SN(%)	SP(%)
GNB-based-wrapper	80.5	0.610	82.8	78.1
DT-based-wrapper	69.2	0.384	68.4	70.0
LongR-based-wrapper	75.4	0.511	80.5	70.4
KNN-based-wrapper	74.6	0.492	72.1	77.1
SVM-Poly-based-wrapper	77.1	0.550	85.5	68.7
SVM-RBF-based-wrapper	80.1	0.605	84.8	75.4
Our method	86.2	0.724	87.2	85.2

### Independent dataset—PDB186

For the purpose of analyzing the robustness, our method is compared to other methods on the independent dateset (PDB186) (PDB1075 serves as training dataset and PDB186 is applied as test dataset), shown in Table 6. Our method achieves 76.34% of ACC, 0.5566 of MCC, 92.5% of SN and 60.22% of SP. Our approach still performs better than most of existing methods with a certain creditability.

**Table 6:** The performance of our method and other existing methods on PDB186 dataset.

Methods	ACC(%)	MCC	SN(%)	SP(%)
IDNA-Prot|dis	72.0	0.445	79.5	64.5
IDNA-Prot	67.2	0.344	67.7	66.7
DNA-Prot	61.8	0.240	69.9	53.8
DNAbinder	60.8	0.216	57.0	64.5
DNABIND	67.7	0.355	66.7	68.8
DNA-Threader	59.7	0.279	23.7	95.7
DBPPred	76.9	0.538	79.6	74.2
iDNAPro = PseAAC-EL	71.5	0.442	82.8	60.2
Kmer1+ACC	71.0	0.431	82.8	59.1
Local-DPP(n = 3,λ = 1)	79.0	0.625	92.5	65.6
Local-DPP(n = 2,λ = 2)	77.4	0.568	90.3	64.5
Our method	76.3	0.557	92.5	60.2

PDB1075 serves as training dataset and PDB186 is applied as test dataset.

### Computational time

The computational time of feature extraction and jackknife test evaluation on PDB1075 is shown in Table 7. From the table, we can find that the computational time of jackknife test evaluation which has used feature selection algorithm is significantly shorter than the jackknife test evaluation without feature selection. This prove that the feature selection algorithm can effectively reduces redundant features.

**Table 7:** The computational time of feature extraction and jackknife test evaluation on PDB1075.

Feature	FE(sec)	JT(sec)	JT-FS(sec)
NMBAC	3.09	2317.6	486.7
DCT	187.98	1357.5	352.0
DWT	299.75	16166.0	757.8
NMBAC+DCT+DWT	490.82	17520.0	1642.0

The values of column “FE” indicate the computational time of feature extraction on PDB1075. The values of column “JT” indicate the computational time of jackknife test evaluation which has not used feature selection algorithm on PDB1075. The values of column “JT-FS” indicate the computational time of jackknife test evaluation which has used feature selection algorithm on PDB1075.

## Conclusion

In this paper, we propose a novel feature extraction algorithm to construct a machine learning method of DNA-binding protein prediction. We employ the feature extraction algorithm to extract three feature vectors, namely NMBAC, PSSM-DWT and PSSM-DCT. It is meaningful that we apply the DWT and DCT methods, which are rarely used in bioinformatics to obtain PSSM-DWT and PSSM-DCT. Through these approaches, the effective information is extracted from the PSSM matrix and stored in the feature vectors. In Jackknife test, our method can achieve excellent prediction performances, and our prediction performance has obviously exceeded other existing methods after feature selection. On the independent dataset, our approach still performs better than most of existing methods. Furthermore, we can find that the PSSM-DWT feature makes the greatest contribution to the prediction performance. The performance of our method proves the rationality of feature extraction algorithm and the effectiveness of our method in predicting DNA-binding protein.
